# Supplementary material for: Are carbon emissions trading and green financial instruments synergistic? -Comprehensive quantitative research based on content analysis
Source: PLoS One. 2024 Mar 7;19(3):e0298601. doi: 10.1371/journal.pone.0298601 (PMC10919601; doi:10.1371/journal.pone.0298601)
Supplement: S1 Data — (ZIP) [file pone.0298601.s001.zip › Data/地级市Z_F工作报告.zip!/║ú─╧/║ú┐┌╩╨/└┤╫╘╣1⁄2╓┌║┼í╛┬φ┐╦╩2╛▌═°í┐_2005.docx]

各位代表：

　　现在，我代表市人民政府，向大会作政府工作报告，请予审议，并请各位政协委员和其他列席人员提出意见。

　　一、２００４年政府工作回顾

　　２００４年，我市坚持以邓小平理论和“三个代表”重要思想为指导，认真贯彻落实中央和省委、省政府的方针政策，以科学发展观统领全局，解放思想，求真务实，开拓进取，扎实工作，圆满完成了市第十三届人大二次会议确定的各项工作任务。

　　经济总量不断壮大。据初步核算，２００４年，全市实现地区生产总值２５３．０１亿元（不含农垦），同比增长１３．２％。其中，一、二、三产业分别增长７．８％、１４．５％和１３．３％。固定资产投资１１９．２８亿元，增长１５．４％。外商直接投资３．２０亿美元，增长１５．６％。财政总收入５１．４４亿元，增长２０．０％；地方一般预算收入１４．９０亿元，增长２１．６％。城镇居民人均可支配收入８９８１元，增长７．６％；农民人均纯收入３５７２元，增长９．２％。整体经济跃上一个新台阶，综合实力和竞争力进一步增强。

　　支柱产业发展加快。工业保持较快发展。“工业发展年”成效明显，海汽三期扩建、药谷核心区建设、三星光纤光缆项目建设、亚太啤酒二期扩建、１６万吨瓶级聚酯切片项目建设等进展顺利，工业规模不断扩大。食品饮料、化纤纺织、烟酒等传统工业也得到较快的发展。全市完成工业增加值５５．２３亿元，增长１４．１％。农业稳步发展。大力推进农村产业结构调整，推广“五统一分”经营模式，加快农业产业化进程；强化农产品安全工作，已获得中国果菜无公害科技示范市县称号，全市无公害蔬菜基地已达７．８万亩；造林２４０１公顷，超额完成省下达的造林任务；全力抓好禽流感预防，确保全市没有高致病性禽流感发生，成功举办海口市无疫区农业发展论坛暨招商会；着手羊山地区开发，促进农村协调发展；积极组织群众抗旱保生产，实现了农业增产、农民增收。全年完成农业增加值２１．３３亿元，增长７．８％。旅游业进一步发展。充分利用“健康岛”、“生态岛”、“无疫区”品牌，加大旅游促销力度，参加上海中国国际旅游交易会、大阪国际旅游展等国内外旅游活动，签署《泛珠三角区域（部分）城市旅游合作协议》，积极开拓客源市场。组织好第二届海口旅游商品展销会、首届中国青年欢乐节暨第五届海南岛欢乐节。全年旅游接待人数４６０．６３万人次，增长１８．２％；实现旅游总收入４２．７６亿元，增长２０．１％。商贸业稳中见旺。新增３０多家大中型网点、近百家连锁点，新增商业经营面积１０万平方米，总投资达１２亿元，南海大道汽车交易一条街等专业市场已初具规模。市场繁荣，销售畅旺。我市被评为全国市场运行监测先进单位。全年完成社会消费品零售总额１０１．０２亿元，增长１７．３％。房地产业持续快速发展。全年房地产开发投资４０．２９亿元，增长４５．４％；房屋施工面积５４７．３２万平方米，增长３６．１％；房屋销售面积１０７．７２万平方米，增长１２．６０％；房屋销售总额２４．１０亿元，增长２０．４％。

　　人居环境不断优化。推行“阳光规划”工程，完成城市总体规划纲要的编制，完成南渡江滨江地带和东海岸城市设计国际招标，开展水系规划和城市公园系统规划，启动城中村改造规划。实施市政工程２５项，其中新开工１６项，续建９项，完成投资４．７亿元。１．３８万亩土地已收归土地储备库。旧城区改造稳步推进，新区配套设施不断完善，人居环境建设进一步加强。创新管理手段，加强城市综合管理，创建先进城市工作成效显著。２００４年我市荣获“中国人居环境奖”，进一步提升了城市的品位和知名度。

　　改革开放取得明显成效。进一步推行政府机构改革，完成国资委、商务局、发展和改革局、人口和计划生育局、安全生产监督局等机构设置和调整。推进城建系统行政管理体制和事业单位机构改革，加快理顺市、区两级管理体制步伐。实行交、巡警合一和城市管理综合执法。以优化产权制度为核心，稳步推进国有企业改革。加快推进开发区改革，全市开发区从２２个压缩到３个。不断完善“政府入口、市场出口”的城市建设投融资体制，对社会投资项目实行核准制和备案制，对政府投资项目实行复审制、回避制和分离制，全面推进部门预算和国库集中收付改革。对外开放上新台阶。继续推行大项目招商战略，在引进大企业、大集团方面取得新的进展。全年共组织参加７次重大招商活动，签约项目１３个，投资额达１２７．７４亿元。

　　社会事业稳步发展。实施技术创新工程，已建立国家级企业技术研究开发中心１个，省级２１个，市级１３个，技术创新示范企业１４家，有力地推动高新技术产业的发展。加强农村实用技术推广，建成农业科技“１１０”服务站８个。着力改善城乡学校的教学条件，投资１９９３万元，完成农村中小学２．８万平方米Ｄ级危房改造；投资２９４５．４万元，完成２８个布局调整项目，撤并调整１５６所农村初小和教学点。全面清理整顿医疗保健服务机构，依法取销１５６家医疗机构执业许可证，医疗药品市场体系进一步完善。全市期末人口出生率１０．３５‰，人口自然增长率７．１４‰，被授予全省人口与计划生育目标管理先进单位称号。文明生态村建设稳步推进，精神文明建设迈上新的台阶。文化、体育、统计、防震减灾、安全生产、外事侨务、双拥、广播电视、妇女儿童、老龄、残疾人等各项社会事业全面进步。

　　群众生产生活条件明显改善。一是切实解决农民的生产生活困难。完成新打井及配套工程２６７宗，改善２８４个村庄、７．０３万人的饮水条件，基本解决我市农村饮水难问题。改建、新建１３条４６．９５公里乡村道路，改善农村交通条件。解决历年拖欠农民征地款４．３亿元的遗留问题。继续组织机关干部深入农村扶贫帮困，受帮扶农村面貌明显改善。二是积极扶持弱势群体。共拨１７２２．７万元，对８万多户（次）城镇困难居民和１１．７万多户（次）农村困难居民进行生活救助；减免城乡特困家庭子女近６０００人的学杂费、课本费２４．７５万元，解决４００多户特困户住房困难问题。千方百计拓宽就业再就业渠道，全年新增就业人员２．８９万人，超额完成省政府下达的任务。三是努力完善社会保障体系。全面实施农村居民最低生活保障制度，全市３万多农村贫困居民纳入最低生活保障，保障金标准从８０元提高到９３元。全年五项社会保险基金征收５．１２亿元，正常支付全市职工医疗、失业、工伤等保险金，按时足额发放３．７９万名离退休人员养老金。积极推进农村新型合作医疗制度，首期试点覆盖８个乡镇２０多万农民，已有１１．４万农民参加合作医疗，占应参加人数的６０．１％。四是全力维护社会稳定。坚持社会治安综合治理的方针，妥善处理各类群体性事件，及时发现与调解各类矛盾和问题，主动、耐心、细致地做好矛盾化解工作，严厉打击各类严重刑事犯罪，维护社会稳定。

　　政府自身建设不断加强。修改地方性法规６件，清理政府规章３４件、行政许可事项３０３项、行政许可实施主体３９个、政府规范性文件１６３件，大大简化了审批程序。开展３个多月的整顿机关作风警示教育，机关作风明显改善，工作人员责任感明显增强。推进机关优质服务活动，着力解决诚信缺失和执行难等突出问题*9熏提高机关服务质量与效率。认真贯彻落实党风廉政建设和反腐败的各项部署，严格执行党风廉政建设和反腐败责任制。认真接受市人大及其常委会的依法监督和政协的民主监督，全年办复市人大代表建议２３３件及政协委员提案２４２件，办结率１００％。完善投资环境监督专员制度，主动接受投资环境监督专员对政府行政的监督。切实贯彻党的十六届四中全会精神，围绕新时期党的中心工作，不断加强行政能力建设。

　　各位代表，过去一年，在省委、省政府和市委的正确领导下，在全市人民共同努力和各方面大力支持下，我们求真务实，开拓进取，抢抓机遇，扎实工作，实现了国民经济持续快速发展和社会全面进步。在此，我代表市政府向全市各族人民、驻市部队、武警官兵、港澳台同胞、海外侨胞、国际友人，向所有关心支持海口建设的投资者和朋友们，表示衷心的感谢并致以崇高的敬意！

　　回顾２００４年，我们也清楚地看到，我市经济社会发展中还存在许多困难和问题。一是产业发展水平仍然较低，经济基础不扎实；二是农业仍然是整个经济的薄弱环节，农民收入不稳定；三是固定资产投资规模偏小，投资结构需进一步优化；四是城市建设、城市管理与省会城市发展的要求还有较大差距；五是社会事业发展相对滞后，城乡就业压力较大，部分群众生活仍然困难；六是政府作风建设、行政效能和投资环境等方面仍有许多不如人意的地方；七是体制创新力度不够，制度建设滞后，国企改革任务繁重，历史遗留问题没有完全解决，社会不稳定因素增多。我们将高度关注这些问题，并在今后的工作中，迎难而上，开拓创新，努力加以解决。

　　二、２００５年主要工作任务

　　２００５年是贯彻落实科学发展观、完成“十五”计划、谋划“十一五”规划的关键一年。目前，我市经济发展景气度不断上升，发展思路更为清晰，人心已拢聚到加快发展上来，内外环境都有利于我市的经济社会发展。根据省委、省政府的总体要求和市委的部署，今年政府工作的总体要求是：坚持以邓小平理论和“三个代表”重要思想为指导，以科学发展观统领经济社会发展全局，围绕提高工业化、城市化水平和加强“三农”工作，大力调整经济结构，培育壮大优势产业，提高经济增长的质量和效益，提升城市核心竞争力，着力推进改革开放，增创体制新优势，完善城市综合功能，优化人居环境，努力构建和谐海口，实现经济社会快速健康协调发展。

　　今年我市经济社会发展的主要预期目标是：地区生产总值增长１３％，地方财政一般预算收入增长１４．７％，固定资产投资增长１５％，社会消费品零售总额增长１１％，城镇居民人均可支配收入增长８％，农民人均纯收入增长７％，城镇登记失业率控制在３．５％以内，人口自然增长率控制在８‰以下。

　　实现上述目标，重点做好以下工作：

　　（一）推动重点项目建设，提高工业化水平

　　要解决我市工业化水平低、经济总量偏小的矛盾，关键在培育和发展一批有市场潜力、支撑海口长远发展的重点项目和支柱产业。要以效益为导向，集中资源重点扶持汽车制造、制药、光纤光缆、化纤纺织、食品饮料、精细化工、热带农副产品深加工等产业。发展循环经济和生态产业，走新型工业化道路。力争今年实现工业总产值２７８亿元，同比增长１５％。

　　加快在建重点项目建设。加快汽车工业发展。尽快落实海汽三期扩建项目征地工作，确保总投资２５亿元的１９个汽车配套项目如期开工，力争使海汽成为年产值超过２００亿元的大型企业。迅速做大药谷。高标准、高水平建设好药谷核心区一期工程，力争万特、海灵化学、信邦、康力元等５家企业投产，力促齐鲁制药等５家企业尽快开工建设；抓紧解决申请二期入驻药谷的１５家企业的用地问题。做强光纤光缆产业。全力做好三星光纤光缆项目的跟踪服务工作。加快发展化纤产业。推动１６万吨瓶级聚酯切片项目建设，扩大化纤产业规模。

　　抓紧招商项目落实。要加强招商项目的筛选，抓好招商引资重点项目的落实，彻底解决招商有项目、落户无土地的问题。对所有签约项目实行“三定”：定项目责任人，定跟踪服务责任，定工作进度。对投资金额较大、带动作用强的重大项目实行市领导跟踪联系制度。其它项目按照“谁签约、谁跟进”和“项目属地管理”的原则，落实到主管部门。及时解决项目建设中需要政府解决的各种问题，真正使重点项目招进来、扎下根、建设快、发展好。

　　培育具有比较优势的项目。今年全省实施的“十项工程”，涉及海口的就有六项。我们必须牢牢抓住这一重大机遇，充分发挥海口资源、人才、产业、环境等优势，积极谋划生态环保、油气和油气化工等领域的项目建设，力争在相关产业方面有所作为。要做好工业西移规划，利用三港合一的契机，引导工业项目向西部转移，逐步建成我市西部邻港工业园区，以港兴市。

　　（二）提高农业综合生产能力，增加农民收入

　　认真贯彻落实今年中央１号文件精神，按照 “城乡互动、三次产业联动”的要求，切实加强“三农”工作。今年要力争完成农业生产总值３７亿元，同比增长８％。

　　加大对农业的政策支持，加快发展现代农业。全面落实免征农业税政策，落实良种补贴和农机具购置补贴政策。加大财政支农力度，拓宽支农资金渠道。以市场为导向，调整优化农业结构，重点发展冬季瓜菜、热带水果、花卉、海淡水养殖、中深海捕捞等，形成“一镇一品”格局。发挥我市“无疫区、无公害”的生态品牌优势，努力扩大畜禽产品的生产，力争今年畜牧业产值占农业总产值的比重达到４０％。建立农产品标准化评价制度和市场准入制度，加快畜禽产品质量安全认证，高标准建设无公害畜禽基地和标准化生产综合示范区，加快农业标准化体系建设。发展壮大一批龙头企业，以企业带基地，以基地带农户，努力构建农工贸、产加销一体化经营体系，推进农业产业化经营。

　　加快农村基础设施建设，重点推进羊山地区发展。按照因地制宜、实用长效的原则，大力实施千里乡村道路建设工程、“５１１工程”（连续５年，每年综合整治１１个大田洋）和文明生态村建设工程，突出抓好农村道路、水利、沼气、供电、广播电视通讯等“五网”建设，进一步改善农民生产和生活条件。今年财政安排２００万元，完成尚未通车的１８个自然村的道路建设，实现我市农村道路村村通的目标。立足羊山地区资源优势，着力发展火山岩特色经济，提高羊山地区发展速度和效益，加快贫困地区脱贫致富步伐，全面推进小康社会建设。

　　发展打工经济，拓宽农民增收渠道。要把农村劳动力转移作为发展经济、脱贫致富的重要措施抓实抓好，抓出成效。建立市、区发展打工经济联席会议制度，积极引导、组织农民外出务工，鼓励发展劳务中介机构，扩大对发达地区的劳务输出规模，提高劳务输出质量和效益。增加投入，加强对农民劳务技能的培训，提高农民就业能力，力争全年完成３万人次的培训、４０００人劳务输出任务。

　　当前，在谋划全年“三农”工作的同时，要充分调动各方面力量，采取有力措施，全力做好抗旱工作，确保全年农业生产有一个良好的开局。

　　（三）引进消费需求，加快发展现代服务业

　　做强旅游娱乐业。做好编制海口市旅游产业发展规划、休闲娱乐业指导目录、旅游业应急预案等基础性工作。创新旅游促销方式，政府的资源主要放在城市形象营销和打造城市名片上，旅游企业的资源则主要放在与资源市场有关的项目上，提升旅游促销整体水平和效能。要统筹全市旅游、休闲娱乐和文化体育资源，统筹旅游和娱乐企业资源，搭建海口市景区管理和旅游基础设施投融资平台。加快景区（点）建设，尽快形成一批能留得住消费者的景点和项目。培育和扶持高尔夫等康体娱乐产业，设立专项资金扶持旅游演艺活动，丰富海口的夜间文化娱乐生活，把海口打造成“娱乐之都”。成立专业节庆会展机构，高水准策划活动，保证每月都有节庆促销活动。强化旅游行业管理，规范旅游市场秩序，积极组织“打造中国旅游名城，开展旅游优质服务年”活动，全方位提供优质服务。今年旅游业完成接待过夜人数力争达到５１０万人次，同比增长１１％；实现旅游总收入４７亿元，同比增长１０％。人均逗留天数和人均消费水平都要有明显增加和上升。

　　做旺商贸业。依托港口、机场、铁路、公路，进一步完善物流服务体系，积极培育连锁经营、集中配送、电子商务等现代物流业。加快城市结构性贸易设施建设，继续抓好南海大道汽车销售一条街、海南ＧＳＰ医药城、海南第一物流中心及建材陶瓷、花卉等批发市场建设。继续完善商业网点规划，重点做好乡镇商业网点和城市新区商业网点发展规划，以及旧城区商业网点改造规划。培育大型商业流通企业集团，鼓励、扶持优势商贸企业加快规模化发展。建立放心安全的市场消费环境。运用市场机制激活消费和引导消费需求，活跃城乡市场。今年社会消费品零售总额要达到１１３亿元。

　　做精房地产业。按照“从严控制、适度发展、限制增量、盘活存量”的原则，以我市荣获“中国人居环境奖”为契机，充分利用得天独厚的居住环境优势，做精房地产业。提高房地产开发的品质。鼓励支持整体规划、成片开发、规模发展，改变房地产小、散、乱的发展格局，使粗放式的房地产发展模式向集约化、精品化、生态化的发展模式转变。加强用地管理。严格依照政策法规，强化土地管理，优化用地结构，制定房地产项目用地的商务条件和技术条件，提高房地产开发项目的审批门槛。严格控制商品房用地的供给，提高土地使用效益，从今年起沿海一线地不再搞商品房开发。创新和完善土地储备机制，加紧依法回收闲置建设用地，增加土地储备量。积极处置半拉子工程，促成１４６项半拉子工程复工建设。加大房地产供给的调控力度，培育大众房地产市场，规范、引导房地产业健康发展。在海口市半拉子工程和闲置楼盘没有消化之前，对商品房批建面积实施总量控制。

　　做活现代服务业。培育和发展城市文化产业、康体保健产业和教育产业。加快文化、体育基础设施建设，做好全民健身中心、体育中心、青少年文化活动中心、歌剧院前期论证等准备工作。积极开发既有地方特色又有一定市场前景的文体产品，创造条件吸引大型文化、体育活动在海口举办，活跃文化体育市场。充分利用全市医疗卫生资源优势和环境优势，吸引国内外资金到海口设立康复疗养基地。积极引进国内外优质教育资源，加快教育产业发展。今年要成立市政府控股的投融资公司，统筹社会领域的政府性投资，加大对上述产业的投资力度。加快发展金融、中介、信息和社区服务等现代服务业。

　　（四）加强城市建设和管理，提高城市化水平

　　在提高产业发展水平的同时，进一步加快我市城市化步伐。要以创建一流人居环境和创业环境为目标，优化城市规划，继续加大城市建设力度，不断完善城市基础设施，完善城市功能，下大力气提高城市管理水平，努力把海口建设成为中心城市功能强、生态环境良好、文化品位高的省会城市。

　　完善城市规划，提升城市品位。今年要建立和完善城市规划的四层次管理体制，成立海口市规划委员会，对重大城市规划项目实行票决制审批。出台海口市城市规划管理标准和技术标准，进一步明确海口城市规划的标准体系、基本色调和建筑风格，推出具有标志性、代表性的城市名片类建筑，提高城市文化含量，提升城市的整体品位。增加绿地面积、增加视觉通道、降低容积率、降低密度，提高城市环境和视觉质量。政府要通过规划把更多的城市空间留给市民，把最好的视觉资源留给百姓，把最适宜休闲旅游的区域让给游客，真正体现以人为本，坚持公共利益至上。

　　推进城市建设，完善城市功能。海口作为省会城市，要积极争取有关部门支持，本着适度超前、量力而行的原则，统一规划和建设城市公共服务设施，不断完善城市综合配套功能。集中财力完成几个久拖未结又直接影响民生的项目建设，减少半拉子工程，确保龙昆南路、南海大道（二期）、城西路、凤翔路改造以及美舍河整治等续建工程按时完工。分清轻重缓急，先办群众最急需的事，尽快打通城市主要通道，完成街巷路灯改造等“民心工程”。严格执行基本建设程序，规范操作，加强业主资格认证，推行工程项目基本建设程序科学管理，强化工程质量监督。坚决做到项目前期准备不充分的不准开工，配套资金不闭合的不准开工，项目和资金管理不规范的不准开工。

　　高效管理城市，优化人居环境。城市管理水平偏低，已经成为影响我市综合发展效益的重要因素和市民关注的热点问题。要下大力抓好城市管理，向管理要效益。重点推进城市综合执法和城市管理重心下移，开展违章建筑整治和交通秩序整治。要创新城市管理手段和办法，从公共基础设施维护、园林绿化、市容管理、交通管理、社区管理等方面入手，制定符合海口市情的城市管理标准化体系，逐步推行规范化、法制化、科学化的城市管理，构建城市管理的长效机制，从根本上改变城市管理混乱的局面。

　　统筹城乡建设，加快城乡一体化进程。要统筹城镇规划，通盘考虑城市建成区与周边中心镇的建设，促进城市建成区基础设施向小城镇延伸，城市公共服务向小城镇覆盖。加大对小城镇、村庄的建设力度，充分发挥小城镇对农村的带动作用。推动农村人口合理有序向集镇、城市转移，加快城市化进程。

　　（五）加大体制创新力度，增强发展动力

　　继续深化各项改革。改革已进入全面攻坚阶段，改革的复杂性和艰巨性增大，我们要以制度创新为根本，深化改革，不断增强发展的动力和活力。本着以存量换增量、以增量促发展和优质资产优先重组的原则，继续深化国有企业产权改革，加快椰岛、椰树及国有控股公司的改制重组，加快城建、交通、公共事业、旅游等系统的国有企业改革，深化国有粮食购销市场化改革，积极引进外资、社会资金或民营企业参与改造、改制和重组。认真贯彻落实《海口市关于加快民营经济发展的决定》，真正实现民营企业与外资和国有企业享受同等机会和市场条件，各部门要多服务少干预，多设路标少设路障，促进民营经济大发展快发展。继续深化行政管理体制改革，推进以人为本的城市社区组织体制改革。推行事业单位全员聘用制，积极稳妥分类推行事业单位机构改革。创新财税管理机制，加强公共财政建设，继续推行收支两条线财政管理，完善部门预算、国库集中收付制度。进一步深化城市基础设施投融资体制改革，推动资金流建设，完善公共设施项目政府投资补偿机制，降低政府负债水平。进一步完善社会投资核准和备案制度，努力保持经济社会发展应有的基本建设投资规模。继续完善和推广“五统一分”经营模式，深化农村经营体制改革。继续推进科技、教育、文化和卫生等社会事业改革，有效调动、整合相关资源，注重发挥市场机制作用，推进社会事业现代化。

　　进一步扩大开放。创新招商引资方法方式，实施重点产业、重点对象、重点区域招商，强化园区的招商载体功能，做好项目储备，重视前期工作，扩大招商引资成效。积极参与泛珠三角等区域合作，加强与港澳等地区的产业互动。利用海南航权开放的契机，加快海口市对外开放步伐。力争全年实现口岸出口３．８亿美元，增长１１％；实际利用外商直接投资３．７亿元，增长１５％。

　　进一步规范市场秩序。加快发展资本、技术、土地、劳动力等要素市场，规范有形建筑市场和房地产市场交易行为。培育各类社会中介组织、行业协会。继续加大食品、药品、农资、劳动力等市场秩序整顿力度，加强社会管理，严厉打击各种形式的商业欺诈、传销、偷逃骗税、非法用工、非法中介行为。加快社会诚信体系建设，建立企业和个人信用服务体系，积极探索建立信用监督和失信惩戒机制，形成遵纪守法、诚实守信的市场环境。

　　（六）推进社会事业发展，构建和谐海口

　　在全力推进经济发展的同时，着力改变社会事业发展滞后的现状，把解决群众的生产生活困难问题摆上更加突出的位置，切实把人民群众的根本利益实现好、维护好、发展好。２００５年，我市社会事业和为民办实事支出的增幅，将高于ＧＤＰ和地方财政收入的增长速度。

　　实施人才强市战略，增强科技创新和服务能力。切实加强我市实用型人才和专家型人才的培养和引进工作，举全市之力，构筑创新人才高地。发挥市科技顾问委员会和院士工作站智力资源优势，加强产学研结合和科技成果产业化，大力推进科技创新，着力培育具有自主知识产权的技术和产品，推动高新技术产业的发展。抓好高新技术产业园区建设，巩固和发展国家８６３计划成果转化基地，用新技术改造提升食品饮料、纺织化纤等传统支柱产业。大力推广农业先进适用技术，加强对农民技术培训，完善农业科技服务“１１０”体系。

　　加快发展文化、教育、卫生等社会事业。城市以文化论输赢。我们要以文化品位来塑造城市形象，展示城市品牌，以文化氛围来凝聚人心，推动城市发展。今年要全面推进城市文化建设，对海口市城市文化的内涵结构、活动形态、空间布局等进行周密的规划。抓好历史文化名城的申报工作。加大对教育的投入，改善薄弱学校办学条件，加快危房改造，促进农村中小学标准化建设；从今年起对我市公办学校义务教育阶段的学生全部免收学杂费，让全市所有贫困家庭子女都能上得起学；推动发展高等教育和成人教育，围绕市场、产业办职业教育，不断提高教育水平和受教育者的素质。推行全民健身活动，丰富城乡群众文化体育生活；有重点地发展竞技体育项目，提高竞技体育水平。认真做好海口医疗卫生规划，优化配置城镇医疗卫生资源，抓紧完成镇卫生院Ｄ级危房改造工作，年内争取完成全市三分之一卫生院的改、扩建任务；抓紧完成市人民医院改造和１２０急救网络建设，启动市疾病控制中心建设；完善卫生监督体系，抓好社区卫生服务网络建设。加快农村卫生事业发展，加大农村公共卫生基础设施投入，探索构建多元化的农村医疗服务体系。加强人口与计划生育工作，保持全省人口与计划生育目标管理先进单位称号。进一步做好民政、双拥、国防、外事侨务和民族宗教等社会各项工作。

　　全面推进精神文明建设。继续实施《公民道德建设实施纲要》，在全社会深入开展社会公德、职业道德、家庭美德教育，着重抓好未成年人思想道德建设，形成平等互爱、团结互助、融洽和谐的社会氛围。今年要完成未成年人活动场所的选址和前期论证工作。以争创全国文明城市为目标，推进文明行业、文明社区、文明生态村创建活动，进一步提高市民文明素质和城市文明程度。积极开展“献爱心”、“送温暖”、“手拉手”、“希望工程”、“扶残助残”等社会公益活动，大力树立创优争先、扶贫济困、见义勇为的良好风尚，倡导科学文明健康的生活方式，坚决破除迷信。

　　做好劳动就业、社会救助和社会保障工作。做好劳动就业、社会救助和社会保障工作是构建和谐海口的前提，也是社会主义市场经济体制下政府义不容辞的职责。要加快就业服务体系建设，加强就业引导、职业介绍和技能培训，办好城乡劳务市场，千方百计促进就业，全年城镇新增就业岗位力争达到２．７５万个。不断完善城乡困难群众社会救助体系，重点解决就业难、就医难、入学难、住房难、养老难等困难问题。加大社会保险费征稽执法力度，进一步扩大社会保险覆盖面，加强对社会保险资金的管理，保持社会保险资金正常支付，不断提高保障水平。继续推进新型农村合作医疗试点，确保参加新型农村合作医疗的农民占应参加人数的７０％，力争覆盖全市。

　　加强生态环境建设和保护。认真实施《海口生态市建设总体规划》和《海口城市绿地系统规划》。实施“蓝天、碧水、安静”三大工程，巩固国家环保模范城市和“一控双达标”成果。采取分区处理污水和生态化处理垃圾的方式，加快城市污水和垃圾处理设施建设。加强南渡江、美舍河、东西湖、红城湖、大同沟等江、河、湖、渠的水体整治，加大演丰镇和三江镇的湿地、东寨港国家级自然保护区等生态脆弱区的生态保护力度，提高生态环境质量。加大“五大道路”出口造林、路边林、村边林建设，增加城市绿化面积。加强地表水饮用水源保护，从重打击非法抽取地下水、乱挖河沙、乱采石、烧山毁林、捕杀珍稀野生动物等违法行为。加强开发建设项目的环境监管，遏制新污染源的产生。

　　大力推进民主法制建设。坚持行政决策科学化、民主化、制度化，积极推行重大决策、重大项目报告制度、论证制度、专家咨询制度和听证制度，广泛听取专家学者、人民群众的意见和建议。实行重要行政事项公示制度，充分保障人民群众知情权、参与权和监督权。认真办理和落实“两会”代表提案、建议和议案，坚持政府向人大报告工作制度，自觉接受人大的依法监督和政协的民主监督。抓好法制宣传教育，增强全民法制观念；继续深入贯彻实施《中华人民共和国行政许可法》，完善各项配套制度建设；积极做好地方政府立法工作，提高制度建设质量。

　　努力维护社会稳定。进一步加强和改进来访来信工作，积极预防和妥善处置群体性事件，及时化解社会不稳定因素。建立社会治安综合治理长效机制，坚决铲除“黄赌毒”社会丑陋现象，严厉打击“两抢”、涉毒、涉枪和暴力犯罪等各类严重刑事犯罪活动，力争各类案件发生率增幅控制在８％以内。建立健全公共突发事件应急机制，加强全民消防安全意识，做好安全生产、交通安全和防火防灾等工作，全力维护社会安定和政治稳定。

　　三、创新政府管理方式，提高行政效能

　　今年是我省“投资环境建设年”。我市要进一步转变政府职能，创新管理方式，强化服务意识，抓好执行和落实，优化投资环境，增强发展优势。

　　转变政府职能，建设公共服务型政府。要改变“以批代管”、“只批不管”的管理方式，加快审批型政府向公共服务型政府转变，把能下放给各区管理的事权交给各区，把不该由政府管、管不了也管不好的事交给企业、市场和社会组织，使政府的职能尽快转变到经济调节、市场监管、社会管理和公共服务上来，把财力物力等公共资源更多地向社会管理和公共服务倾斜，把领导精力更多地放在促进社会事业发展和建设和谐社会上。行政审批实行目录化管理，执行同一审批标准，增强透明度，并逐步由“指令性”管理转变为“禁令性”管理。尽快发挥行政服务中心的作用，减少审批事项，简化审批程序，实行审批事项的内容、程序、权限、时限、收费、结果“六公开”。

　　加快制度建设，提高政府管理社会水平。政府对人民权利的保护是最好的公共服务，政府要公开承诺公共服务的质量。政府的统筹决策要事先进行可行性研究，对成本和效益进行估算。重大决策项目要有告示制度，建立、健全有社会各方面人士参加的咨询委员会和政策听证制度，充分发挥新闻媒介的信息披露作用。建立以公共服务为取向的政府业绩评价体系和行政问责机制，欢迎社会各界以社会发展指标考核政府的政绩。认真落实市政府近期颁布的《海口市人民政府会议制度》、《政府投资项目管理规定》、《海口市城市规划审批管理规程》等规范性文件，从制度上进一步规范政府的行政行为，防止和减少行政过错。

　　加强政风建设，优化行政环境。牢固树立科学发展观和正确政绩观，大兴求真务实之风。坚决反对形式主义和虚报浮夸，不搞劳民伤财的“形象工程”、“政绩工程”。减少会议和文件，改进会风和文风。以贯彻《海南省行政首长问责暂行规定》为契机，进一步加大对投资环境特别是政务环境的监督力度，对决策失误、违法行政、执行不力、疏于管理等问题进行问责；对行政不作为和乱作为、效率低下、办事推诿的部门和工作人员，将采取严厉的措施。谁破坏海口发展环境，谁将付出沉重代价。设立市长接待日制度，听取企业、市民对政府工作的意见和建议。认真贯彻中纪委五次全会和国务院第三次廉政工作会议精神，全面推进廉政建设和反腐败工作。结合开展保持共产党员先进性教育活动，加强对公务员的教育、管理和监督，努力建设一支勤政务实、廉洁高效的人民满意的公务员队伍。

　　推进信用建设，提高政府公信力。要增强承诺要兑现、决策要执行、办事要落实的信用意识，不折不扣地完成政府承诺的为民办实事的各项工作，真正取信于民。加强对政府债务、行政遗留问题和政府投资建设项目遗留问题的清理，并千方百计逐步加以解决，真正取信于社会。加快推行行政承诺、行政过错补偿、基础设施建设项目依时竣工等制度，切实维系政府的公信度。

　　各位代表，展望未来，我们肩负着更加光荣而艰巨的使命。让我们紧密团结在以胡锦涛同志为总书记的党中央周围，在省委、省政府和市委的领导下，高举邓小平理论和“三个代表”重要思想伟大旗帜，全面落实科学发展观，团结一致，开拓创新，求真务实，扎实工作，为建设海口更加美好的明天而努力奋斗！
